# Complete Genome Sequence of Human Oral *Actinomyces* sp. HMT897 Strain ORNL0104, a Host of the Saccharibacterium (TM7) HMT351

**DOI:** 10.1128/MRA.00040-21

**Published:** 2021-04-08

**Authors:** Karissa L. Cross, Floyd Dewhirst, Mircea Podar

**Affiliations:** a Biosciences Division, Oak Ridge National Laboratory, Oak Ridge, Tennessee, USA; b Department of Biological Sciences, Vanderbilt University, Nashville, Tennessee, USA; c Department of Microbiology, The Forsyth Institute, Cambridge, Massachusetts, USA; University of Rochester School of Medicine and Dentistry

## Abstract

*Actinomyces* sp. HMT897 strain ORNL0104 was isolated from a human saliva sample and can serve as a host for the ectobiont *Saccharibacteria* (TM7) HMT351. Its 3.3-Mb chromosome was completely sequenced using PacBio long reads, and it encodes 2,407 proteins and 63 RNAs.

## Abstract (precis)

*Actinomyces* sp. HMT897 strain ORNL0104 was isolated from a human saliva sample and can serve as a host for the ectobiont *Saccharibacteria*/TM7 HMT351. Its 3.3-Mb chromosome was completely sequenced using PacBio long reads, and it encodes 2,407 proteins and 63 RNAs.

## ANNOUNCEMENT

*Actinomyces* (phylum *Actinobacteria*) is a genus of bacteria that colonize the human body, primarily the oral cavity. There are at least two dozen formally described human-associated species; some still await validation, and others have not yet been cultured (1, 2). While the relative abundance of some *Actinomyces* spp. can change across different oral environments and between health and disease (gingivitis, periodontitis, caries), where they may be implicated in the etiology of those conditions, *Actinomyces* spp. can also cause specific infections (actinomycosis) at other body sites (1, 3–5). Several oral *Actinomyces* spp. have been shown to be hosts for ectobiotic *Saccharibacteria* (TM7) (6–10). *Actinomyces* sp. HMT897 strain ORNL0104 was coisolated with the saccharibacterium (TM7) HMT351 (10), and it represents an as yet undescribed species. Here, we report its complete genome sequence.

*Actinomyces* sp. HMT897 strain ORNL0104 was grown anaerobically (90% N_2_, 10% CO_2_) in brain heart infusion (BHI) medium (Difco) for 2 days at 37°C. Genomic DNA was isolated using a proteinase K-SDS digestion and phenol-chloroform extraction protocol detailed in reference 17. DNA sizing using the Femto Pulse system (Agilent) revealed a peak at 11 kb with fragments extending to >50 kb. A library was prepared using the SMRTbell template prep kit v1.0 (Pacific Biosciences, Menlo Park, CA). Sequencing was performed in multiplex mode on a Pacific Biosciences Sequel instrument using a Sequel II 8M single-molecule real-time (SMRT) cell. Default parameters were used with all the sequence analysis software unless otherwise specified. Sequence quality-based filtering and assembly were performed using HGAP4 in PacBio SMRTLink v7, with a target genome size of 3 Mbp, minimum read mapping length of 500 nucleotides (nt), and minimum mapping concordance of 70. A total of 219,932 polymerase reads (mean length, 45,463 nt) and 1,033,094 subreads (*N*_50_ length, 10,329 nt) were assembled in two contigs (2.78 Mb and 538 kb) with mean confidence (Phred quality value, QV) of 88 and 2,575-fold mean coverage. The contig ends were trimmed (20 to 30 kb on each end), where coverage was >500-fold, and were assembled in a single contig with Geneious Prime v2021.0.1 (11). We performed gene calls and annotation using Prokka v1.14.0 (12). Analysis of the annotated contig ends revealed identical regions, and the nucleotide position unique for each end was identified. Based on that, the ends were joined in Geneious, resulting in a circular genome sequence of 3,281,265 nt with a G+C content of 71.6%.

**FIG 1:**
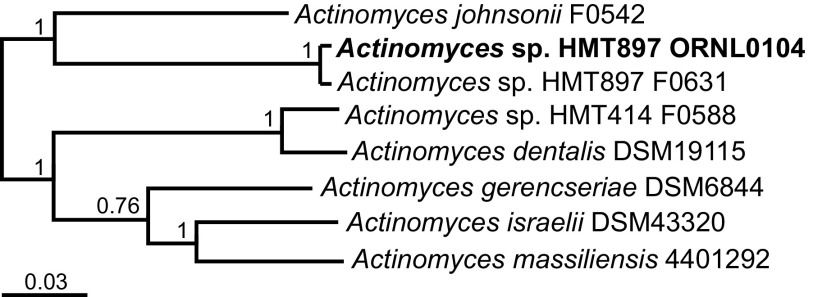
Phylogenetic tree of *Actinomyces* sp. HMT897 strain ORNL0104 and related human oral *Actinomyces* spp. based on 49 core, universal bacterial proteins, using KBase-FastTree2 (16). Numbers at the nodes indicate support values.

We used the NCBI Prokaryotic Genome Annotation Pipeline (PGAP) v4.8 (13) for final gene prediction and annotation. The *Actinomyces* sp. HMT897 strain ORNL0104 chromosome encodes 2,407 proteins, 51 tRNAs, 3 rRNA operons, and 3 noncoding RNAs (ncRNAs). The genome was compared with those of other human *Actinomyces* strains using FastANI v0.1.2 and SpeciesTreeBuilder v1.0 in KBase (14). Based on an average nucleotide identity (ANI) value of 98.3% (15) between strain ORNL0104 and another HMT897 strain, isolated at the Forsyth Institute (F0631, GenBank accession number CP027236), both belong to the same, as yet undescribed, species of *Actinomyces*. *A. johnsonii* F0542 is the closest described species to the two HMT897 strains (ANI, 80.6%) (Fig. 1). The genome will be useful for studying the diversification of oral *Actinomyces* spp. in the context of health and disease as well as the evolution and physiology of *Actinomyces*-*Saccharibacteria* (TM7) relationships.

### Data availability.

The *Actinomyces* sp. HMT897 strain ORNL0104 genome sequence has been deposited in GenBank under the accession number CP067336. The version described in this paper is the first version, CP067336.1. The PacBio reads have been deposited in the SRA under the accession number SRX9831799.
